# RNA-binding protein RNPC1: acting as a tumor suppressor in breast cancer

**DOI:** 10.1186/1471-2407-14-322

**Published:** 2014-05-07

**Authors:** Jin-Qiu Xue, Tian-Song Xia, Xiu-Qing Liang, Wenbin Zhou, Lin Cheng, Liang Shi, Ying Wang, Qiang Ding

**Affiliations:** 1 Jiangsu Breast Disease Center, the First Affiliated Hospital with Nanjing Medical University, 300 Guangzhou Road, Nanjing 210029, China

**Keywords:** RNPC1, Breast cancer, p53, EMT, Tumor suppressor

## Abstract

**Background:**

RNA binding proteins (RBPs) play a fundamental role in posttranscriptional control of gene expression. Different RBPs have oncogenic or tumor-suppressive functions on human cancers. *RNPC1* belongs to the RNA recognition motif (RRM) family of RBPs, which could regulate expression of diverse targets by mRNA stability in human cancer cells. Several studies reported that RNPC1 played an important role in cancer, mostly acting as an oncogene or up-regulating in tumors. However, its role in human breast cancer remains unclear.

**Methods:**

In the present study, we investigated the functional and mechanistic roles of RNPC1 in attenuating invasive signal including reverse epithelial-mesenchymal transition (EMT) to inhibit breast cancer cells aggressiveness *in vitro*. Moreover, RNPC1 suppress tumorigenicity *in vivo*. Further, we studied the expression of RNPC1 in breast cancer tissue and adjacent normal breast tissue by quantitative RT-PCR (qRT-PCR) and Western blot.

**Results:**

We observed that RNPC1 expression was silenced in breast cancer cell lines compared to breast epithelial cells. More important, RNPC1 was frequently silenced in breast cancer tissue compared to adjacent normal breast tissue. Low RNPC1 mRNA expression was associated with higher clinical stages and mutp53, while low level of RNPC1 protein was associated with higher lymph node metastasis, mutp53 and lower progesterone receptor (PR). Functional assays showed ectopic expression of RNPC1 could inhibit breast tumor cell proliferation *in vivo* and *in vitro* through inducing cell cycle arrest, and further suppress tumor cell migration and invasion partly through repressing mutant p53 (mutp53) induced EMT.

**Conclusions:**

Overall, our findings indicated that RNPC1 had a potential function to play a tumor-suppressor role which may be a potential marker in the therapeutic and prognostic of breast cancer.

## Background

Breast cancer is the most commonly diagnosed cancer in women and the leading cause of cancer deaths in the developed world [1]. Despite advances to diagnose and treat breast cancer keeping growing, the incidence is still rising, and it remains a major fatal disease in women [2]. Breast cancer is a heterogeneous disease due to complicated etiology, results from accumulated genetic and epigenetic alterations of various cancer genes, including tumor-suppressor genes (TSGs) and oncogenes [3]. RNA binding proteins (RBPs) have been realized as novel layer of gene regulation and involved in breast cancer progression as TSGs or oncogenes.

RBPs play a key role in posttranscriptional control of gene expression [4], such as polyadenylation, RNA splicing, transport, stability, and translation, all of which are emerging as critical mechanisms for gene regulation in mammalian cells [5]. RBPs contain one or more RNA-binding motifs, such as hnRNP K homology motif, RNA recognition motif (RRM), RGG box, and dsRBD motif [5-7]. RRM is the most prevalent type of eukaryotic RNA-binding motifs [6]. RBPs are involved in the expression of various genes responsible for biological processes and cellular functions, so expected mutations or aberrant production of RBPs can cause cancer progression [7,8]. Deregulation of splicing factors might lead to alternative splicing of transcripts in cancer cells [9]. On the other hand, translation of mRNA is also a regulatory point for the expression of tumor suppressors or oncogenes in cancer cells [10]. Therefore translation factors play critical role in tumorigenesis. Translation initiation factor could be over-expressed in various tumor and behave as a characteristic proto-oncogene [4].

*RNPC1* gene is located on chromosome 20q13 and expressed in various tissues. It belongs to the RRM family of RBPs, is expressed as RNPC1a with 239 amino acids and RNPC1b with 121 amino acids [11]. RNPC1a is capable of regulating biological characteristics, binding and stabilizing the mRNA of p21, p73 and Hu antigen R (HUR) [11-13]. Recently, RNPC1 is also found to bind and stabilize the mRNA of Macrophage inhibitory cytokine-1 (MIC), which facilitates RNPC1-induced cell growth suppression [14]. Additional mRNAs bound by RNPC1 include p63, murine double minute-2 (MDM2) and p53 mRNAs. In these instances, RNPC1 binding mediates a decrease in mRNA levels and attenuation of translation [15-17]. It is solidly confirmed that RNPC1 play pivotal roles in regulating wide biological processes, ranging from cell proliferation, cell cycle arrest to cell myogenic differentiation [13,18]. However, its role in tumorigenesis is scanty and contradictory in human cancers, particularly in breast cancer. In many studies, RNPC1 was recognized as an oncogene, frequently amplified in prostate cancer [19,20], ovarian cancer [21], colorectal cancer [22,23], chronic lymphocytic leukemia [24], colon carcinoma [25], esophageal cancer [26], dog lymphomas [17], and breast cancer [27,28]. Recently, new evidence suggested RNPC1 might act as a tumor suppressor. It was reported to be in a negative feedback loop, which restricts E2F1 activity by limiting cell-cycle progression at the G1-S boundary [29]. Expression of RNPC1 is highly correlated with increased survival in human ovarian cancer [29]. Moreover, RNPC1 was silenced by promoter hypermethylation in breast cancer [30]. However, most of the available studies focused on the various targets of RNPC1 binding in cancer. Its expression and biologic functions in human breast cancer remains unclear.

In this study, we showed that RNPC1 was significantly down-regulated in high-invasive breast cancer cell lines, MDA-MB-231 and SUM1315, not low-invasive MCF-7 cell lines. RNPC1 potentiated tumor-suppressive signals to suppress proliferation, growth, migration, and invasiveness of breast cancer cells *in vitro*, and suppress tumorigenicity *in vivo*. Importantly, we examined RNPC1 expressive situation in clinical cancer and adjacent normal breast specimens and analyzed the association with between RNPC1 expression and clinic pathological characters. RNPC1 was found to be lower expressed in breast cancer compared to adjacent normal breast tissue. RNPC1 mRNA expression was associated with clinical stages and mutp53. RNPC1 protein expression was associated with lymph node metastasis, mutp53 and progesterone receptor (PR). The clinical data was consistent with the experimental results; both of them strongly suggested that RNPC1 might act as a tumor suppressor in breast cancer.

## Methods

### RNA extraction, reverse transcription and quantitative RT-PCR (qRT-PCR)

Total RNA was extracted from cells and tissues using Trizol reagent (TaKaRa, A-79061), and cDNA was synthesized using Primescript RT Reagent (TaKaRa) following manufacturer's instructions. The following PCR primers were used:

RNPC1 forward, 5′-ACGCCTCGCTCAGGAAGTA3-′

RNPC1 reverse, 5′-GTCTTTGCAAGCCCTCTCAG3-′

β-actin forward, 5′-GCTGTGCT ATCCCTGTACGC3-′

β-actin reverse, 5′-TGCCTCAGGGCAGCGGAACC3-′

qRT-PCR for β-actin and other genes was performed for every cDNA sample. All PCR reactions were performed using the fluorescent SYBR Green I methodology. Real-time quantitative PCR was performed on StepOne Plus Real-Time PCR system (Applied Biosystems, USA) using FastStart Universal SYBR Green Master (Roche, Switzerland) according to the manufacturer's instructions. As a result, the relative gene expression was normalized, with β-actin serving as the internal control. Noticeable, this study showed clearly RNPC1instead of RNPC1a.

### Tissue samples

121 pairs of snap-frozen breast tumor and matched normal tissues from adjacent regions were provided by the First Affiliated Hospital with Nanjing Medical University from February 2006 to August 2009, from patients treated surgically for clinical stage I–III breast cancer (aged 34–82 years). All the patients did not receive chemotherapy, radiotherapy or hormone therapy before surgery. Tumor and normal tissue samples had been verified as tumor or non-tumor by histopathological examination of hematoxylin stained paraffin sections. Histologic types were classified according to the World Health Organization (2003). TNM staging was defined according to the American Joint Committee on Cancer (AJCC) (the 6th version, 2002). All the cases were individually categorized by independent pathologists. All the samples’ collection was according to the ethical guidelines of the Declaration of Helsinki and approved by the ethics and research committee of the First Affiliated Hospital of Nanjing Medical University. Before surgery patients are informed that their surgical specimens would possibly be used for research purposes. All the participants provided their written informed consent for inclusion in the data analysis and manuscript publication. Data were analyzed anonymously.

### Cell culture

The human breast cancer cell lines (MCF-7, MDA-MB-231, BT474 and ZR-75) and non-malignant breast epithelial cells (MCF-10A) were obtained from the American Type Culture Collection (ATCC, VA, USA) and culture in complete medium of High glucose Dulbecco's Modified Eagle Medium (DMEM) supplemented with 10% fetal bovine serum (FBS), 1% penicillin - streptomycin solution at 5% CO_2_ and 37°C incubator. Cell line SUM1315 was provided by Stephen Ethier (University of Michigan). The 184A1 immortalized breast epithelial cell line was provided by Ceshi Chen (Kunming Institute of Zoology).

### Plasmid construction and lentivirus packaging

Lentivirus packaging cells were transfected with PGLV3-h1-GFP-puro vector (GenePharma, Shanghai, China) or pGLV5-h1-GFP-puro vector (GenePharma, Shanghai, China) containing either the RNPC1a knockdown (shRNPC1a) or RNPC1a overexpression (RNPC1a), and a scrambled sequence (SCR) or a negative control sequence (NC), respectively, following the manufacturer’s instructions. Three shRNA plasmids (sh1, sh2, sh3) were constructed against different RNPC1a targets, including a scrambled sequence as a negative control (Additional file 1: Table S1). All plasmids were verified by sequencing (GenePharma, Shanghai, China). Cells were plated in 6 wells dishes at 30% confluence and infected with the retroviruses. Meanwhile, polybrene (5 μg/ml) was added with the retroviruses to enhance the target cells infection efficiency. Stable pooled populations of breast cancer cells were generated by selection using puromycin (2 μg/ml) for 2 weeks. For knockdown, one construct (sh3), with ≥85% knockdown efficiency, was used for further studies (Additional file 1: Figure S1).

### Colony formation assay

Cell used for colony formation analysis were seeded into 6-well plates (500 cells/wells) and cultured normally for 15–20 days. The colonies were fixed in paraform and stained with Giemsa after washed by phosphate-buffered saline (PBS) twice, then dried at room temperature. The colonies in each well were counted, and all cell colonies contained 50 or more cells.

### Cell counting kit (CCK-8) assay

Cell proliferation was assessed using CCK-8 kit (Dojindo, Japan) according to the manufacturer’s instructions/protocol. Cells diluted serum-free medium, 2,000 cells/wells were seeded in a 96-well cell culture plate, grown at 37°C on the day of measuring the growth rate of cells, 100 μl of spent medium was replaced with an equal volume of fresh medium containing 10% CCK8, then cells continued to be incubated at 37°C for 3 h, and the absorbance was finally determined at 450 nm using a micro plate reader (5082 Grodig, Tecan, Austria).

### Wound healing assay

Breast cancer cells were seeded into 6-well plates, and allowed to grow until 100% confluens. Then the cell layer was gently scratched through the central axis using a sterile plastic tip and loose cells were washed away. Quantification of cell motility by measuring the distance between the invading fronts of cells in three random selected microscopic fields (200×) for each condition and time point (0, 18 h).

### Cell migration and invasion assays

*In vitro* cell migration and invasion assays were performed as described previously [31]. Images of three random fields (200×) were captured from each membrane, and the number of migratory or invasive cells was counted.

### Tumorigenesis in nude mice

BALB/C female nude mice (4-6-weeks old, 18–22 g) were randomly divided into two groups (each containing 7 mices). Stable RNPC1a-expression MDA-MB-231 cells or control cells (1 × 10^6^ cells in 0.1 ml PBS) was subcutaneously orthotopically injected into mammary fat pads of the mice and the growth of tumors was followed up for 6 weeks. Tumor volume was measured weekly using a caliper, calculated as (tumor length × width^2^)/2. After 6 weeks, mice were sacrificed and checked for final tumor size. Mouse studies were conducted according to the Guide for the Care and Use of Laboratory Animals and approved by the Animal Care and Use Committee of Nanjing Medical University. All the samples’ collection was according to the ethical guidelines of the Declaration of Helsinki and approved by the ethics and research committee of the First Affiliated Hospital of Nanjing Medical University.

### Western blotting analysis

Western blot analysis was performed as described previously [32]. The primary antibodies used were anti-rabbit RBM38 (Santa Cruz), p21 (Santa Cruz), p53 (Santa Cruz), p53 (Millipore), Vimentin (Abcam), anti-mouse E-cadherin (Abcam). The secondary antibodies were purchased from Cell Signaling technology. The intensity of the bands was determined using densitometric analysis. GAPDH (Santa Cruz) was used to as loading control.

### DNA histogram analysis

Cell cycle was assessed by flow cytometry (Becton Dickinson, San Jose, CA, USA). For cell cycle analysis, cells were collected, washed with PBS and fixed in ethanol at −20°C for 8 h before being collected by centrifugation. Then cells were washed with PBS, and resuspended in 500 μl of PBS with 0.2% Triton X-100, 10 mM EDTA, 100 μg/ml RNase A, and 50 μg/ml propidium iodide (PI) at room temperature for 30 min.

### Statistical analysis

The data were analyzed using the SPSS 12.0 software (SPSS, Chicago, IL, USA). All experiments in this study were repeated in triplicate, unless otherwise specified. Student t-test was used to analyze the statistical significance of the differences between groups. χ^2^ test and Fisher Exact test were used to assess the correlation between *RNPC1* and clinicopathologic parameters. For all the tests *p* values < 0.05 was considered statistically significant.

## Results

### RNPC1 was lower expressed in human breast cancer cells

**Figure 1:**
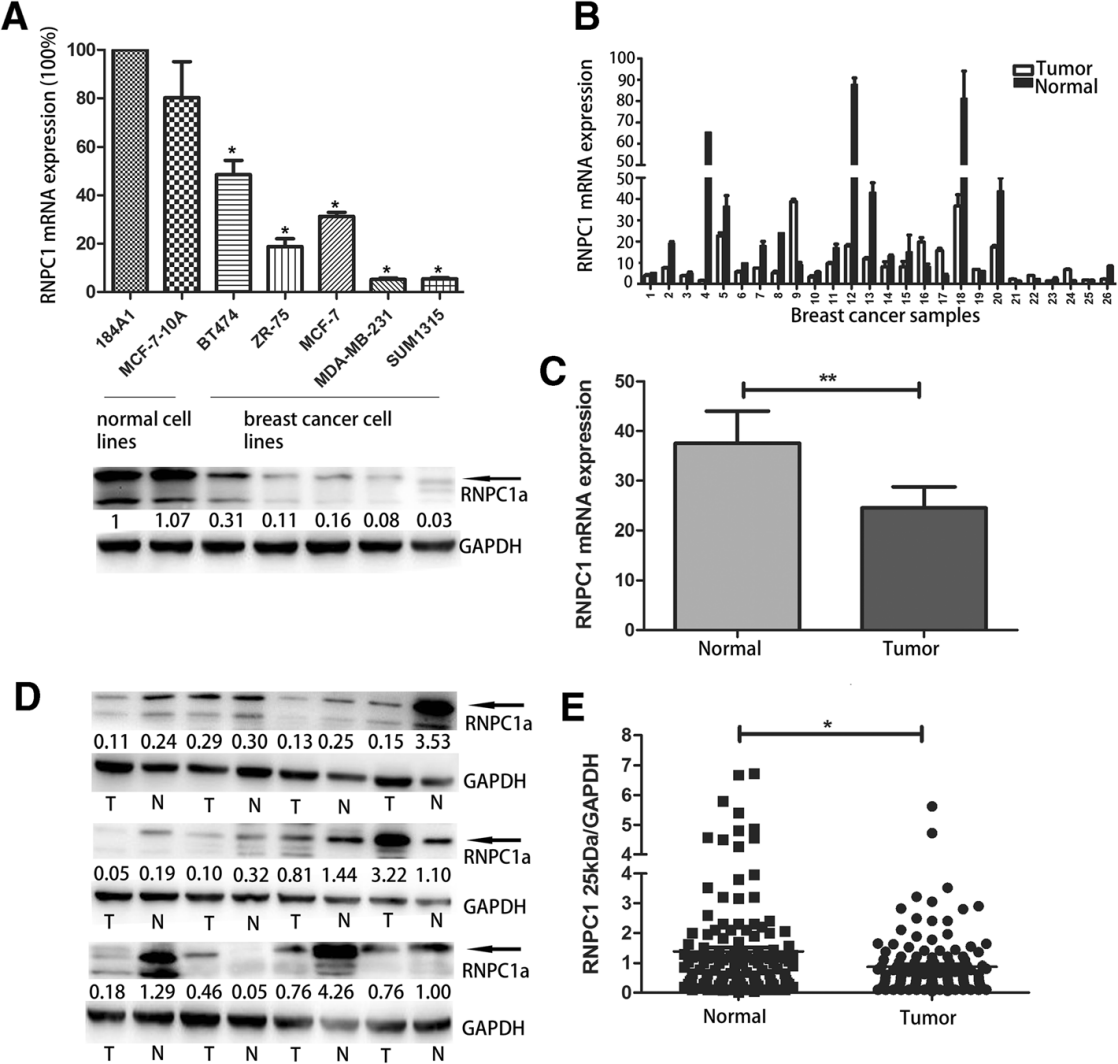
**RNPC1 expressive in breast cancer cell lines and tissues. (A)** qRT-PCR and Western blot analysis of RNPC1 expression in breast cancer cell lines and normal breast cell lines MCF-10A and 184A1. The two normal breast cell lines showed higher expression of RNPC1 than other cell lines (*p* < 0.05). The fold change of RNPC1a is shown *below* each lane. The intensity of the bands was determined using densitometric analysis. **(B)** RNPC1 mRNA expression in 121 pairs of breast cancer and adjacent tissue. 26 cases showed for example. **(C)** Average expression level of RNPC1 mRNA in 121 pairs of human breast cancer tissues and adjacent normal breast tissues. Adjacent breast tissues had higher expression of RNPC1, where the breast cancer tissues showed the lower level of expression (*p* < 0.01). **(D)** RNPC1 protein expression in 121 pairs of breast cancer and adjacent tissues. The 25 kDa band/GAPDH ratio was markedly lower in tumors compared to adjacent normal tissues. 12 cases showed for example. The fold change of RNPC1a is shown *below* each lane. The intensity of the bands was determined using densitometric analysis. **(E)** A scatter plot of RNPC1 protein expression in the same cancer tissue, adjacent tissue (*p* < 0.05). Data were means of two separate experiments mean ± SEM, *p < 0.05, **p < 0.01.

RNPC1 expression in five breast cancer cell lines and two breast epithelial cell lines were quantified by qRT-PCR and Western blot (Figure 1A, *p* < 0.05). Among the seven cell lines analyzed, RNPC1 was found lower expression in breast cancer cells compared to normal mammary breast epithelial MCF-10A and 184A1 cells. Among breast cancer cells, MCF-7, BT474, ZR-75 cells expressed relatively higher levels of RNPC1, and low expression or barely detectable levels were found in MDA-MB-231, SUM1315. Noticeable, this study showed that clearly MB-231 could instead of MDA-MB-231.

### RNPC1 protein and mRNA expression were down-regulated in human breast cancer tissue

To determine RNPC1 expression in breast cancer tissues, we use qRT-PCR and Western blot to analyze mRNA and protein of RNPC1 in 121 breast cancer tissues and marched adjacent non-cancerous tissue. RNPC1 transcripts were expressed at varying levels in the primary breast tumors analyzed. We determined a gene expression cut-off value of 0.61 (median value) that differentiated between RNPC1 low expression and high expression in breast cancer. Similar to the cell lines’ data, of the 121 paired samples, 82 (68%) showed significantly lower RNPC1 mRNA expression in the breast cancer tissue compared to the adjacent tissue. Partial data was showed in Figure1B and 1C (*p* < 0.01), mean level of RNPC1 in tumors and tumor-adjacent normal tissue was 24.52, 37.58, respectively, which suggested that down-regulation of RNPC1 was common in breast cancer. In Western blot analysis, 84 (69%) patients showed significantly lower RNPC1a expression in the breast cancer tissue compared to the adjacent normal tissue, partial data was showed in Figure 1D. The comparison obtained by calculating the ratio between RNPC1a and GAPDH expression (Figure 1E, *p* < 0.05) also showed RNPC1a expression in tumors was lower than the adjacent tissues (mean: 0.87, 1.37). Table 1 displayed the association of RNPC1 expression level and clinicopathological features of 121 breast cancer patients, which demonstrated that low RNPC1 mRNA expression was significantly associated with advanced clinical stages (*p* = 0.010), mutp53 (*p* = 0.042). In addition, it was related with lymph node metastasis (*p* = 0.058) and grade (*p* = 0.066). There was no significant correlation between RNPC1 mRNA expression and patient age, tumor size, Ki67, PCNA, CK5/6, histology, estrogen receptor (ER), progesterone receptor (PR) status or human epidermal growth factor receptor 2 (HER2). Table 2, showed that RNPC1 protein expression was significantly associated with lymph node metastasis (*p* = 0.024), mutp53 (*p* = 0.039) and PR (*p* = 0.023). There was no significant correlation between RNPC1 protein expression and patient age, advanced clinical stages, Ki67, PCNA, CK5/6, histology, or ER status and HER2.

**Table 1:** The association between RNPC1 mRNA expression and clinicopathologic features of breast cancer

**Clinicopathologic parameters**	**Number of case**	**RNPC1**	**RNPC1**	***p*-value**
		**Low expression**	**High expression**	
**Age (years)**				
**≤55**	68	42	26	0.109
**>55**	53	40	13	
**Tumor Size (cm)**				
**≤2**	38	24	14	0.463
**>2**	83	58	25	
**TNM stage**				
**I**	24	11	13	**0.010**
**II + III**	97	71	26	
**Lymph node metastasis**				
**≤3**	96	69	27	0.058
**>3**	25	13	12	
**Grade**				
**I**	7	2	5	0.066
**II**	67	50	17	
**III**	30	18	12	
**unclear**	17	12	5	
**ER status**				
**Negative**	40	22	18	0.097
**Positive**	70	51	19	
**unclear**	11	9	2	
**PR status**				
**Negative**	58	42	16	0.294
**Positive**	63	40	23	
**Her2 status**				
**Negative**	81	56	25	0.298
**Positive**	32	19	13	
**suspect**	8	7	1	
**Ki67**				
**≤15%**	43	30	13	0.430
**>15%**	52	40	12	
**CK5/6**				
**Negative**	46	35	11	0.830
**Positive**	15	11	4	
**P53**				
**Negative**	42	26	16	**0.042**
**Positive**	52	42	10	
**PCNA**				
**Negative**	2	1	1	0.567
**+**	36	27	9	
**++**	22	18	4	
**+++**	12	8	4	
**Histology**				
**ductal**	106	71	35	0.622
**special**	15	11	4	

*ER*: estrogen receptor; *PR*: progesterone receptor; *HER2*: human epidermal growth factor receptor 2.

TNM classification according to the Union Internationale Contre le Cancer criteria. HER2 positivity 3+ in immunohistochemistry or positive fluorescent in situ hybridisation test.

**Table 2:** The association between RNPC1 protein expression and clinicopathologic features in breast cancer

**Clinicopathologic parameters**	**Number of case**	**RNPC1**	**RNPC1**	***p*-value**
		**Low expression**	**High expression**	
**Age (years)**				
**≤55**	68	46	22	0.631
**>55**	53	38	15	
**Tumor Size (cm)**				
**≤2**	38	25	13	0.557
**>2**	83	59	24	
**TNM stage**				
**I**	24	15	9	0.411
**II + III**	97	69	28	
**Lymph node metastasis**				
**≤3**	96	62	34	**0.024**
**>3**	25	22	3	
**Grade**				
**I**	7	4	3	0.215
**II**	67	51	16	
**III**	30	20	10	
**unclear**	17	9	8	
**ER status**				
**Negative**	40	24	16	0.181
**Positive**	70	53	17	
**unclear**	11	7	4	
**PR status**				
**Negative**	58	46	12	**0.023**
**Positive**	63	38	25	
**Her2 status**				
**Negative**	81	61	20	0.117
**Positive**	32	18	14	
**suspect**	8	5	3	
**Ki67**				
**≤15%**	43	30	13	0.402
**>15%**	52	32	20	
**CK5/6**				
**Negative**	46	29	17	0.800
**Positive**	15	10	5	
**P53**				
**Negative**	42	32	10	**0.039**
**Positive**	52	29	23	
**PCNA**				
**Negative**	2	2	0	0.619
**+**	36	27	9	
**++**	22	16	6	
**+++**	12	7	5	
**Histology**				
**ductal**	106	75	31	0.397
**special**	15	9	6	

*ER*: oestrogen receptor; *PR*: progesterone receptor; *HER2*: human epidermal growth factor receptor 2.

TNM classification according to the Union Internationale Contre le Cancer criteria. HER2 positivity 3+ in immunohistochemistry or positive fluorescent in situ hybridisation test.

### RNPC1a inhibited proliferation and growth in human breast cancer cells *in vitro*

**Figure 2:**
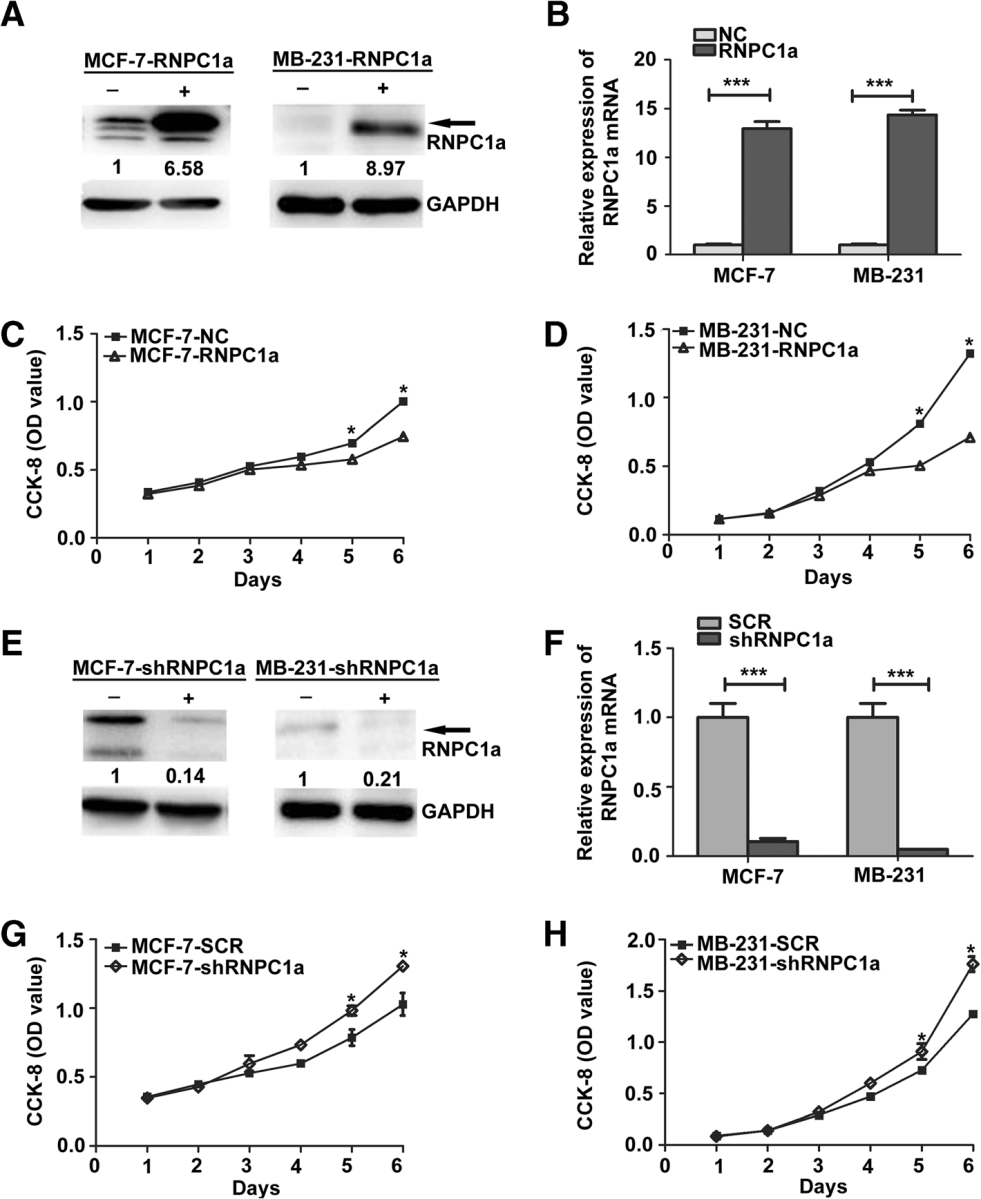
**Effect of RNPC1a on proliferation and growth of breast cancer cell lines MCF**-**7 and MB**-**231. (A)** Western blot and **(B)** qRT-PCR were used to verify the efficiency of RNPC1a overexpression. The fold change of RNPC1a protein is shown *below* each lane. The intensity of the bands was determined using densitometric analysis. **(C,D)** The growth of cells over 6 days was measured using cell counting kit (CCK-8) assays. RNPC1a indicates RNPC1a overexpressing MCF-7 and MB-231 cells; NC indicates MCF-7 and MB-231 cells transfected with a vector-expressing GFP. The proliferation rate of MCF-7-RNPC1a and MB-231-RNPC1a was significantly decreased compared with control cells, respectively. Data were means of three separate experiments mean ± SEM, *p* < 0.05. **(E)** Western blot and **(F)** qRT-PCR were used to verify the efficiency of RNPC1a-knockdown. The fold change of RNPC1a protein is shown *below* each lane. The intensity of the bands was determined using densitometric analysis. **(G**, **H)** MCF-7-shRNPC1a and MB-231-shRNPC1a were significantly increased compared with control cells, respectively. Data were means of three separate experiments mean ± SEM,*p < 0.05, ***p < 0.001.

To further address the functions of RNPC1 in breast cancer cells, we infected MCF-7 cells and MDA-MB-231 cells and selected stably infected cells. The over-expressed cell lines were named as MCF-7-RNPC1a or MB-231-RNPC1a, while the matched control cell lines were named as MCF-7-NC or MB-231-NC, respectively. The silenced cell line was named as MCF-7-shRNPC1a or MB-231-shRNPC1a, while the matched control cell lines were named as MCF-7-SCR or MB-231-SCR, respectively. We confirmed the expression levels using both Western blot (Figure 2A and E) and qRT-PCR (Figure 2B and F, both *p* < 0.001).

**Figure 3:**
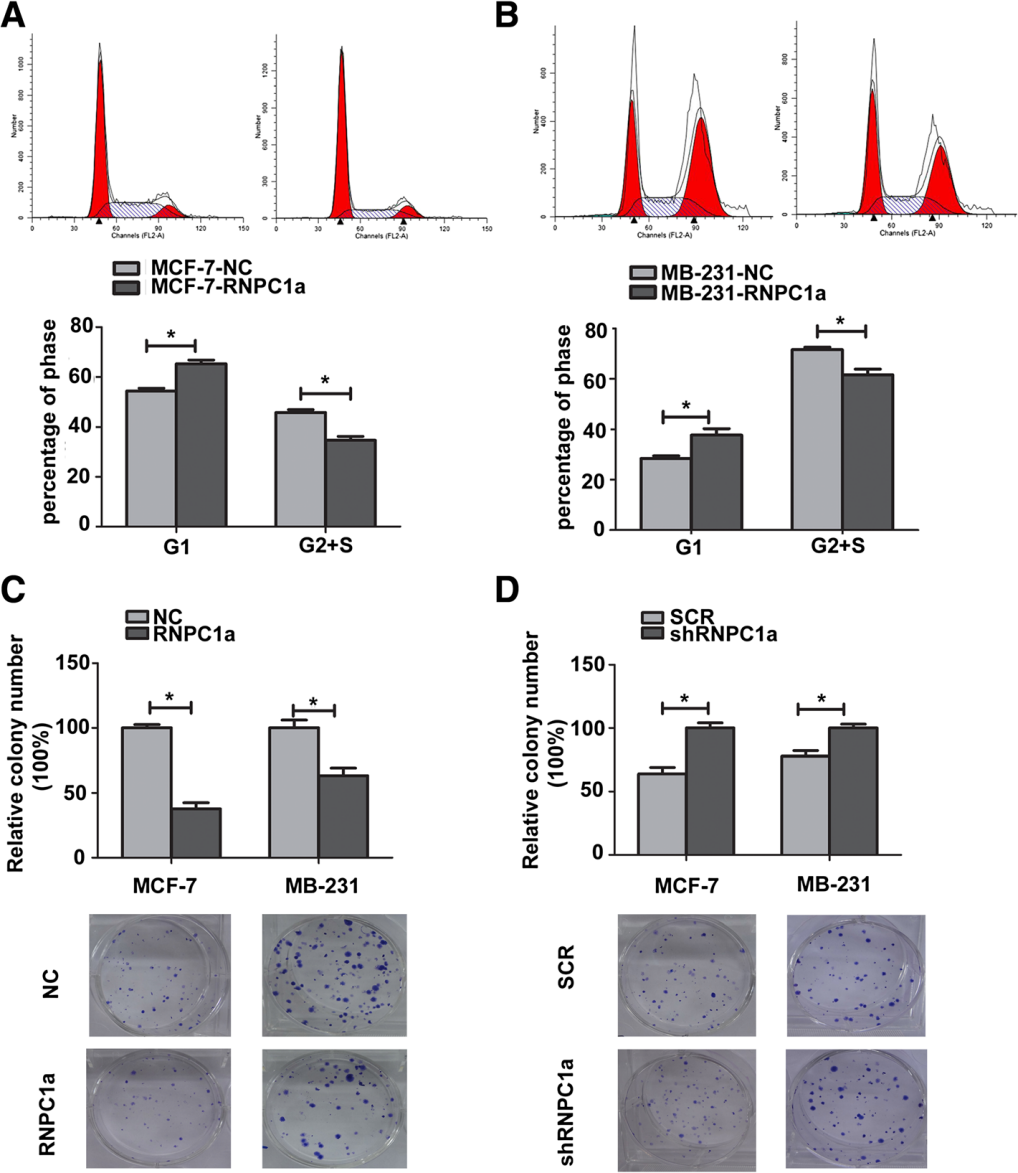
**RNPC1a suppressed anchorage dependent growth of breast cancer cells. (A,B)** Cell cycle progression was measured using flow cytometry. The progression of MCF-7-RNPC1a and MB-231-RNPC1a cells was arrest in the G1 phase compared with control cells, respectively. Representative photographs (upper) and quantification (lower) are shown. **(C)** The growth of cells over 15 days was measured using colony formation assays. Clone formation of RNPC1a overexpression arbitrarily set at 100% in control cells (NC). The number and size of MCF-7-RNPC1a or MB-231-RNPC1a was significantly decreased compared to control cells, respectively. Representative photographs (lower) and quantification (upper) are shown. Data were means of three separate experiments mean ± SEM, *p* < 0.05. **(D)** Clone formation of RNPC1a knockdown arbitrarily set at 100% in knockdown (shRNPC1a) cells. The number and size of MCF-7-shRNPC1a or MB-231-shRNPC1a was significantly increased compared with control cells, respectively. Representative photographs (lower) and quantification (upper) are shown. Data were means of three separate experiments mean ± SEM, *p* < 0.05. Colonies > 50 mm were counted. Anchorage–dependent growth assays were shown at the bottom. Data were means of three separate experiments mean ± SEM, *p < 0.05.

The growth of the stable cell lines over 6 days was determined using Cell counting kit (CCK-8) assay. As shown in Figure 2C and Figure 2D, RNPC1a overexpression led to significantly decreased cell proliferation (*p* < 0.05), while RNPC1a knockdown led to significantly increased cell proliferation (Figure 2G and H, both *p* < 0.05). To further study the mechanism by which RNPC1a overexpression or knockdown affected proliferation, cell cycle progression was analyzed using flow cytometry. MCF-7-RNPC1a cells showed a delayed G1 phase compared to MCF-7-NC cells (65.28 ± 1.495 vs 54.28 ± 1.121) (Figure 3B, *p* < 0.05), while MB-231-RNPC1a cells also showed a delayed G1 phase compared to MB-231-NC cells (37.74 ± 2.559 vs 28.44 ± 1.033) (Figure 3B, *p* < 0.05). RNPC1a overexpression inhibited the proliferation of breast cancer cells via a delay in cell cycle progression. We obtained the similar results in RNPC1a knockdown MCF-7 (Additional file 2: Figure S2).

Since anchorage-independent growth is strongly correlated with tumorigenicity [33]. The ability of MCF-7 or MB-231 cell lines to form colonies was much fewer when RNPC1a was over-expressed (Figure. 3C, *p* < 0.05). The ability of MCF-7 or MB-231 cell lines to form colonies was much more when RNPC1a was knockdown (Figure. 3D, *p* < 0.05).

### RNPC1a suppressed migratory and invasive potential

**Figure 4:**
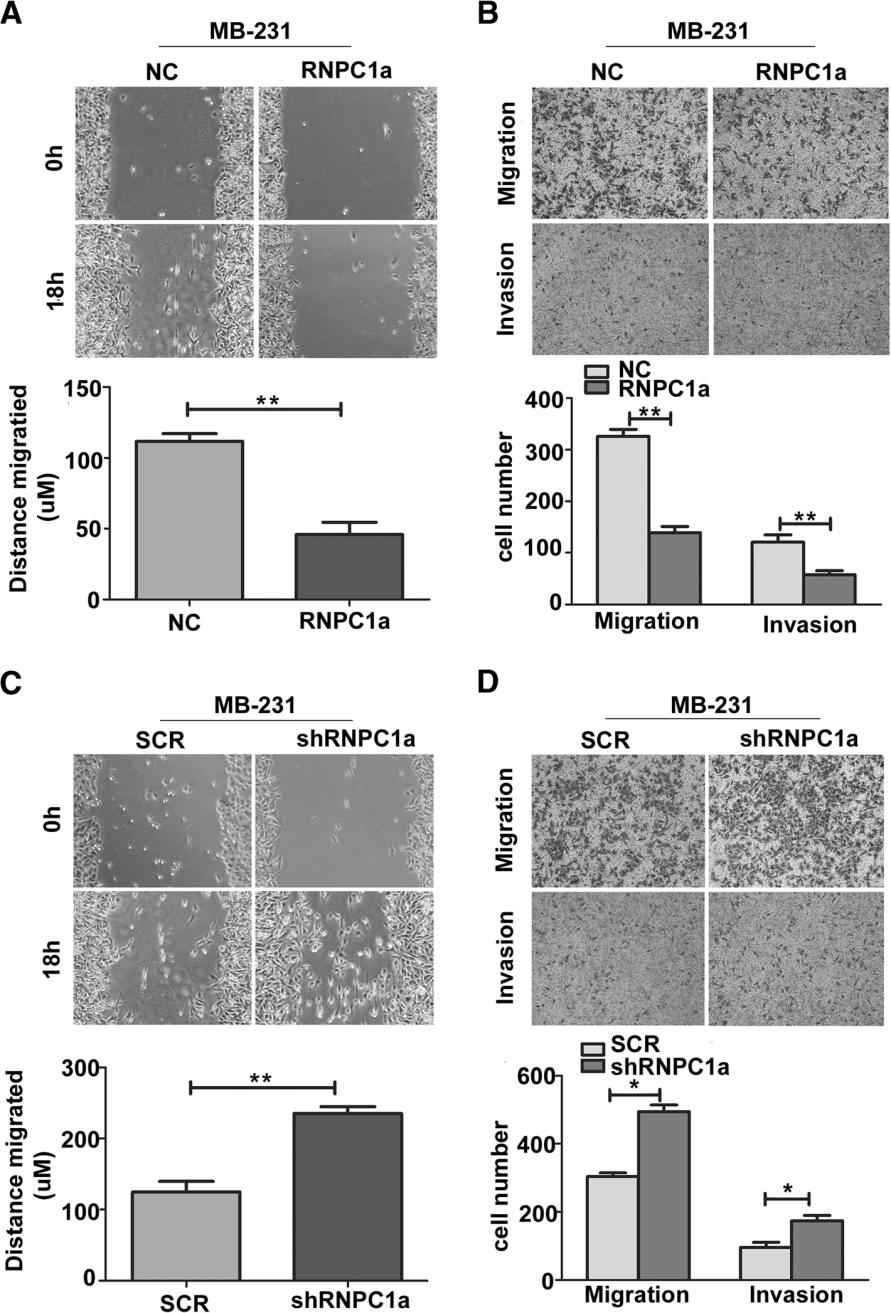
**RNPC1a significantly decreased migratory and invasive potential of breast cancer cells. (A,C)** Wound healing assay. Images of wound repair were taken at 0, 18 h after wound. The distance of wound closure is shown by area at 18 h. Representative photographs (upper) and quantification (lower) are shown, original magnification, ×200. **(B,D)** Transwell migration assay and Matrigel invasion assay. Representative photographs (upper) and quantification (lower) are shown. Columns: average of three independent experiments, *p < 0.05, **p < 0.01, original magnification, ×200.

As shown in Figure 4A and C, determined by their migration in the wound gap after 18 h, distance migrated of RNPC1a overexpression decreased by 69 μm (Figure 4A, *p* < 0.01), while RNPC1a knockdown increased by 110 μm (Figure 4C, *p* < 0.01) compared to the control cells, respectively. We conducted three-dimensional cell migration assay using transwell chambers and invasion assay with Matrigel-precoated transwell chambers. We found that RNPC1a overexpression exhibited significantly decrease ability of migration and invasion (Figure 4B, both *p* < 0.01). RNPC1a knockdown exhibited significantly increase ability of migration and invasion (Figure 4D, both *p* < 0.05). Besides, we obtained the similar results of MCF-7 cells (Additional files 3: Figure S3).

### RNPC1a down-regulate mutp53 and up-regulate p21 protein expression in breast cancer cells

**Figure 5:**
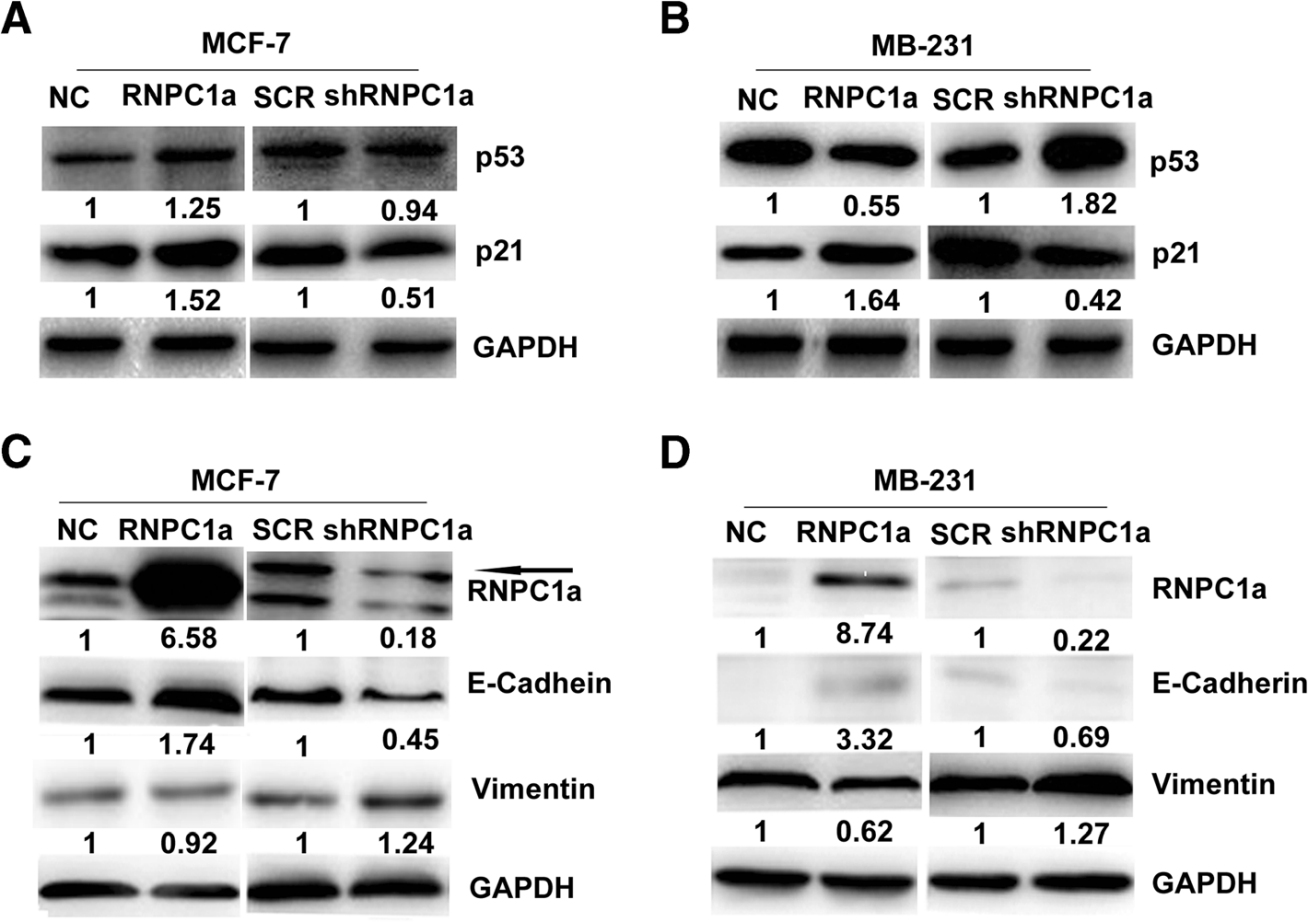
**RNPC1a regulated p53**, **p21**, **E**-**cadherin and vimentin in breast cancer cell. (A)** RNPC1a positively regulated p21, while there was no significantly correlation was found between RNPC1a and wtp53 in MCF-7 cells. **(B)** RNPC1a positively regulated p21, while negatively regulated mutp53 in MDA-MB-231 cells. **(C)** RNPC1a positively regulated E-cadherin, while negatively regulated Vimentin in MDA-MB-231 cells. **(D)** RNPC1a positively regulated of E-cadherin, while there was no significantly correlation was found between RNPC1a and Vimentin in MCF-7 cells. The fold change of RNPC1a is shown *below* each lane. Arbitrarily set at 1.0 in control cells. The intensity of the bands was determined using densitometric analysis.

Previous study affirmed that translational of wild-type p53 (wtp53) was repressed by RNPC1a [17]. However, our study found wtp53 protein was no significantly altered in RNPC1a over-expressed or silent MCF-7 cells (Figure 5A). Level of p21 protein was increased in RNPC1a over-expressed MCF-7 and MDA-MB-231 cells (Figure 5A and B). Mutp53 protein was decreased in RNPC1a over-expressed MDA-MB-231 cells. When RNPC1a was silenced, p21 protein was decreased in MCF-7 and MDA-MB-231 cells, while mutp53 was increased (Figure 5B).

### RNPC1a up-regulate E-cadherin and down-regulate vimentin protein expression in breast cancer cells

We observed that the RNPC1a knockdown in MCF-7 cells led to a spindle-shaped fibroblastic morphology. This morphological change might suggest the phenotypic change of EMT. In addition, RNPC1a over-expressed MDA-MB-231 cells lost their fibroblast-like morphology, which was accompanied by a cobblestone-like epithelial morphology (data not shown). As shown in Figure 5C and Figure 5D the levels of E-cadherin expression was increased in the RNPC1a over-expressed cells, while decreased in the RNPC1a knockdown cells. The levels of Vimentin expression was increased in the RNPC1a knockdown MDA-MB-231 cells, while decreased in the RNPC1a over-expressed cells in MDA-MB-231. But MCF-7 cells were not obviously changed in the protein level of the mesenchymal markers such as Vimentin.

### RNPC1a suppressed tumorigenesis in nude mice

**Figure 6:**
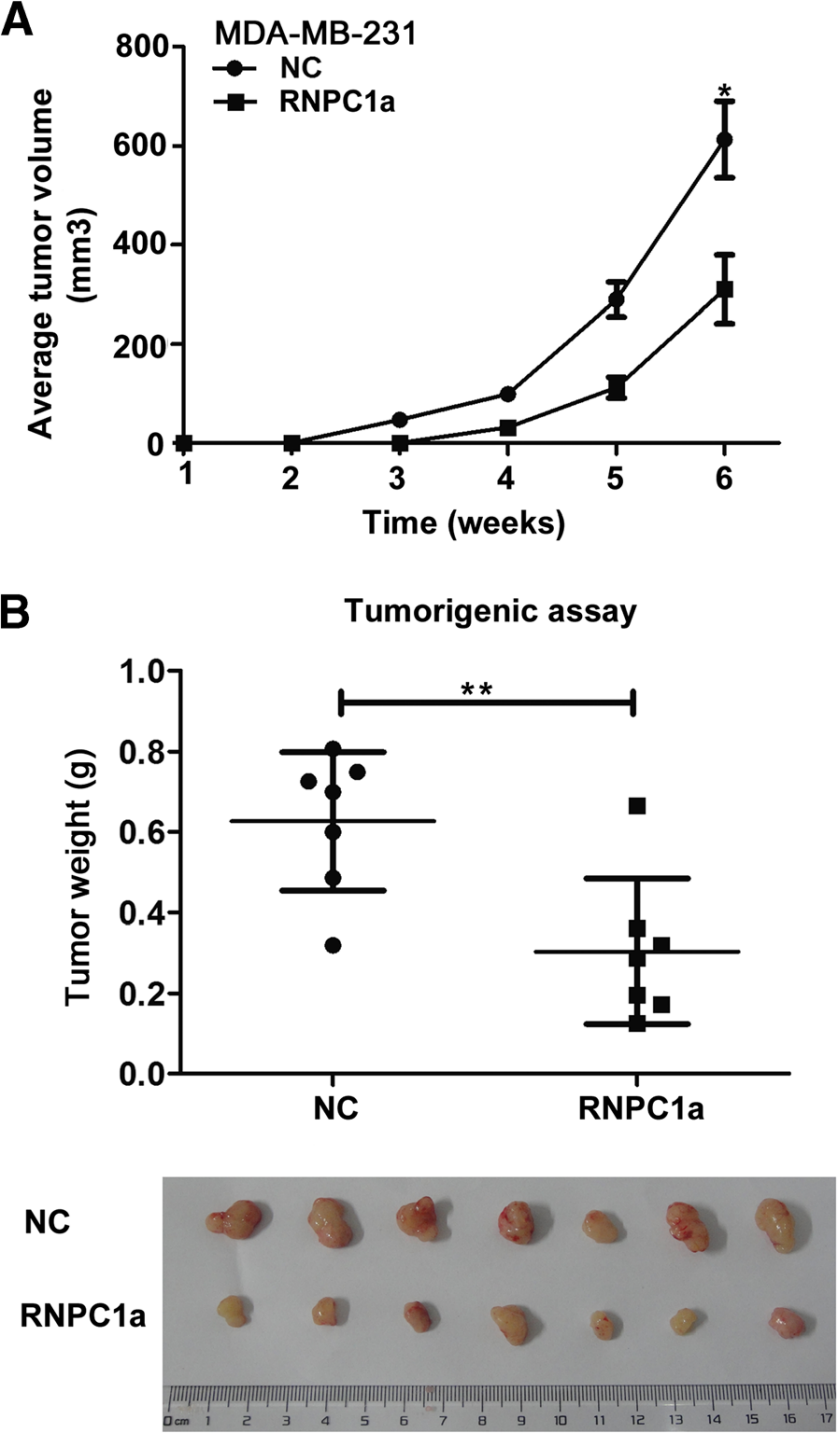
**RNPC1a suppressed tumor growth in nude mice. (A)** RNPC1a over expressed (RNPC1a) MDA-MB-231 cells formed smaller tumor volume compared to the control cells (NC). **(B)** RNPC1a overexpression reduced tumor weight compared to the control cells (NC). Data were means of experiments mean ± SEM, *p < 0.05, **p < 0.01.

To evaluate the tumor-suppressive functions of RNPC1a *in vivo*, tumorigenicity of MDA-MB-231 cells expressing RNPC1a was evaluated in nude mice. Over-expressed RNPC1a and control cells were injected into mammary fat pads of the mice. Control cells were discovered tumors after 2 weeks, while tumors derived from over-expressed RNPC1a cells were discovered after 4 weeks (Figure 6A, *p* < 0.05). RNPC1a over-expressed cells formed smaller tumor volume and weight compared to the control cells (Figure 6B, *p* < 0.01).

## Discussion

This study focused on the biological functions of RNPC1 and its potential clinical value in breast cancer. Among the seven breast cell lines analyzed, RNPC1 was found to be lower expressed in breast cancer cells compared to breast epithelial cells. It implied a suppressive function of RNPC1 in breast cancer. Consistent with this, overexpression of RNPC1 could reduce, whereas knockdown of RNPC1 could accelerate growth rate and number of colonies formation of breast cancer cells. In cancer, proliferation is mostly driven by altered cell cycle progression, apoptosis, or both [34]. Other studies reported that overexpression of RNPC1 could induce cell cycle arrest in G1 in colon cancer RKO [11] and osteosarcoma U2OS [29]. Cell cycle arrest in G1 was also observed in RNPC1 expressed breast cancer cells. Conversely, RNPC1 knockdown induced cell cycle progression. Meanwhile, we observed that p21 was changed concomitantly with RNPC1, suggesting RNPC1 in part induce cell cycle arrest in G1 via binding to and stabilizing p21 transcript [11,35]. Further *in vivo* data finally supported the suppressive function of RNPC1 in breast cancer cells. RNPC1 over-expressed MDA-MB-231 formed smaller tumor in nude mice compared to the control cell. These results were well consisted with experimental data both *in vitro* and *in vivo*.

Motility and invasion are the major events in metastasis of cancer [36]. In recent years, epithelial-mesenchymal transition (EMT) has been proved to be an important step during the progress from primary tumor to metastases [37,38]. EMT is required for normal mammary gland development [39] and breast cancer progression [40]. Among the tested cell lines, RNPC1 barely expressed in mesenchymal phenotype breast cancer cell lines compared to the epithelial breast cancer cell lines. We also observed that MDA-MB-231 cells lost their fibroblast-like morphology, when RNPC1 was over-expressed, and transformed to a cobblestone-like epithelial morphology. Inspiring of that, we supposed that RNPC1 might inhibit migration and invasion of breast cancer cells by regulating EMT. By regulating RNPC1 expression, we found E-cadherin was promoted in the RNPC1 overexpression breast cancer cell lines, whereas Vimentin level was reduced.

Mutations of p53 tumor suppressor was often highly expressed and has a long half-life in various tumor [41]. Mutp53, is the commonest genetic variation detected in primary breast cancer [42], which has various types of functions requiring therapeutic targeting [43]. Former study found RNPC1 and wtp53 were negative feedback loop [17]. RNPC1 overexpression inhibited mutp53 in colon cancer [17]. We first reported that RNPC1 overexpression decreased mutp53 protein expression in breast cancer. Mutp53 could induce partial EMT-like transitions reflected in the ability to suppress E-cadherin synthesis [44,45]. It implied that mutp53 may participate in RNPC1 regulated process of EMT. The mechanisms about how RNPC1 regulate transcriptional factors to inhibit EMT are requiring more investigation in the future.

Based on clinical samples, we observed that RNPC1 was widely expressed in non-cancerous normal breast tissues but frequently down-regulated in breast cancer tissue, consisting with the *in vitro* data. The clinic RNPC1 low expression may be explained by promoter hypermethylation correlates with wtp53 status [30]. By analyzing the clinic data from 121 pairs of specimens, we found significantly negative correlation between RNPC1 mRNA expression and mutp53, clinical stages; significantly negative correlation between RNPC1 protein expression and lymphonode metastasis; significantly positive correlation between RNPC1 protein expression and PR. The same trend was also found between RNPC1 and mutp53protein. These results were well consisted with experimental data both *in vitro* and *in vivo*.

Since RNPC1 is one of p53’s targets, the level of RNPC1 in breast cancer may depend on the p53 status. Thus, it is also possible that the correlation between RNPC1 mRNA and clinical stages actually represents the correlation between the p53 status and clinical stages. Just like ER regulating PR pathway, and both of them making the most important molecular markers of breast cancer, RNPC1 could develop to a novel molecular maker as a downstream factor of p53.

## Conclusions

In summary, RNPC1 was frequently loss or low-expressed in breast cancer. RNPC1 inhibits breast cancer cells proliferation and further suppressed tumor-cell migration and invasion. RNPC1 significantly negative correlated between RNPC1 protein and mutp53, lymphonode metastasis, clinic stage. It suggested RNPC1 acting as a functional tumor suppressor in breast tumorigenesis and metastasis. RNPC1 may become a potential marker in the therapeutic or prognostic practice of breast cancer.

## Competing interests

The authors declare that have no competing interests.

## Authors’ contributions

QD and J-QX have contributed to the conception and design of the study, T-SX performed the analysis and interpretation of data, as well as final approval of the version to be submitted. X-QL and WZ participated in the design of the study, performed the statistical analysis, drafted and revised the article. LC, LS, YW performed the experimental study. All authors read and approved the final version of manuscript.

## Pre-publication history

The pre-publication history for this paper can be accessed here:

http://www.biomedcentral.com/1471-2407/14/322/prepub

## Supplementary Material

Additional file 1: Table S1 RNPC1a shRNA sequences. **Figure S1.** Identification of stably transfected MCF-7 and MB-231 cells. (A, C) Western blot was used to verify the efficiency of knockdown. The cells transduced with the three shRNAs and one control shRNA are designated as ‘sh1’, ‘sh2’, ‘sh3’ and ‘SCR’. RNPC1a-knockdown MCF-7 and MB-231 cells had 85% lower expression when compared with SCR cells. The fold change of RNPC1a is shown *below* each lane. Arbitrarily set at 1.0 in control cells. The intensity of the bands was determined using densitometric analysis. (B, D) qRT-PCR was used to detect RNPC1a expression. The results are similar to those seen in the Western blot analyses. Data were means of two separate experiments mean ± SEM, * *p* < 0.05. Click here for file

Additional file 2: Figure S2 Cell cycle was progress in RNPC1a knockdown MCF-7 cells. (A) The progression of MCF-7-SCR cells was more arrested in the G1 phase compared to MCF-7-shRNPC1a cells. (B) Histogram of cell cycle analyses. Data were means of three separate experiments mean ± SEM, *p < 0.05. Click here for file

Additional file 3: Figure S3 RNPC1a decreased migration and invasion in MCF-7 cells. (A) The number of migrating and invading cells was higher in MCF-7-NC than the MCF-7-RNPC1a cells. (B) The number of migrating and invading cells was lower in MCF-7-SCR than the MCF-7-shRNPC1a cells. Data presented average number of cells/field for three fields. (C, D) Columns: average data of three independent experiments, mean ± SEM, *p < 0.05, ***p < 0.001. Click here for file
