# Supplementary figures and images for: RNA-binding protein RNPC1: acting as a tumor suppressor in breast cancer
Source: BMC Cancer. 2014 May 7;14:322. doi: 10.1186/1471-2407-14-322 (PMC4101826; doi:10.1186/1471-2407-14-322)

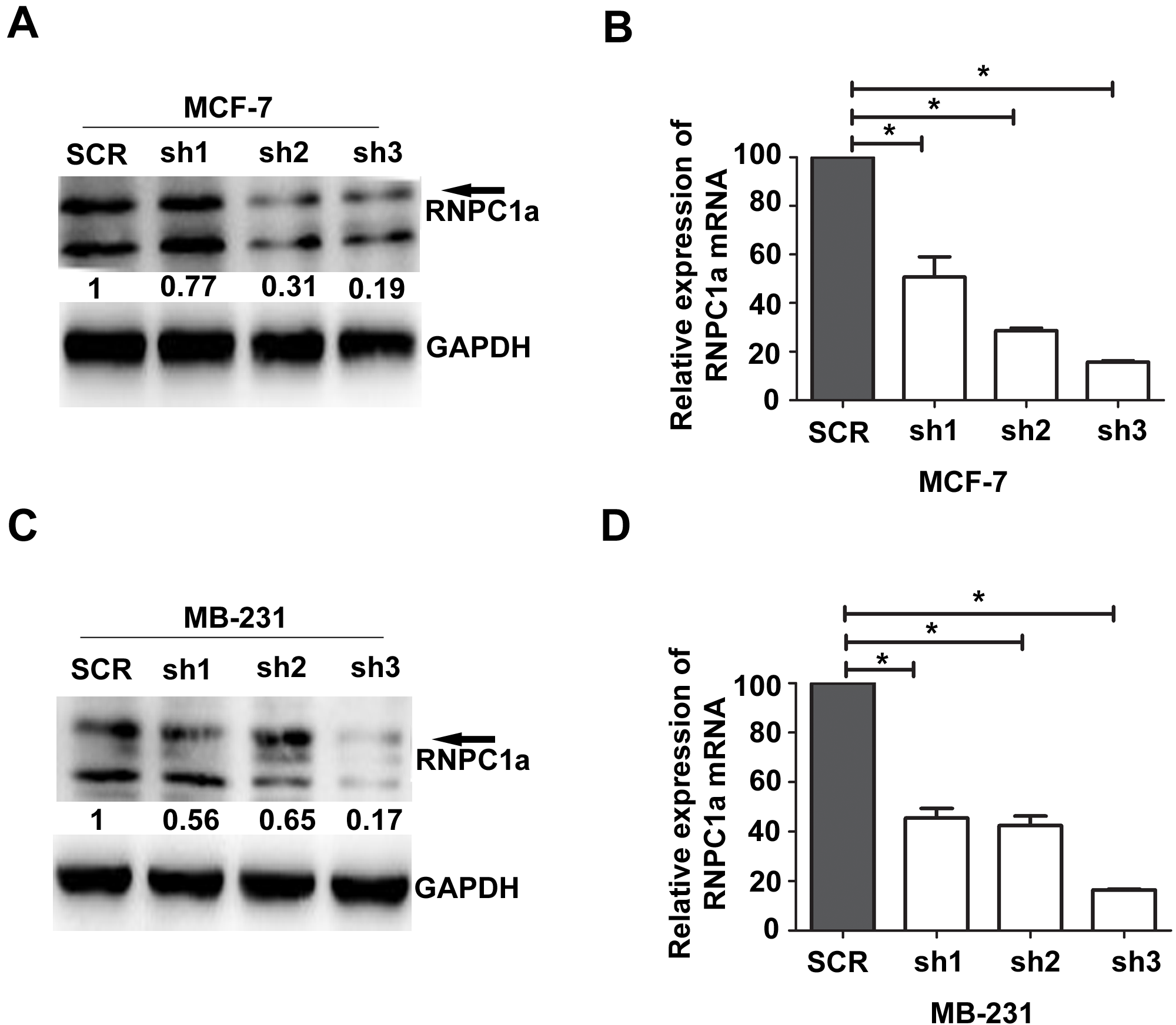

Supplement: Additional file 1: Table S1 — RNPC1a shRNA sequences. Figure S1. Identification of stably transfected MCF-7 and MB-231 cells. (A, C) Western blot was used to verify the efficiency of knockdown. The cells transduced with the three shRNAs and one control shRNA are designated as ‘sh1’, ‘sh2’, ‘sh3’ and ‘SCR’. RNPC1a-knockdown MCF-7 and MB-231 cells had 85% lower expression when compared with SCR cells. The fold change of RNPC1a is shown below each lane. Arbitrarily set at 1.0 in control cells. The intensity of the bands was determined using densitometric analysis. (B, D) qRT-PCR was used to detect RNPC1a expression. The results are similar to those seen in the Western blot analyses. Data were means of two separate experiments mean ± SEM, * p < 0.05. [file 1471-2407-14-322-S1.zip › Additional file 1/Figure S1.tif]

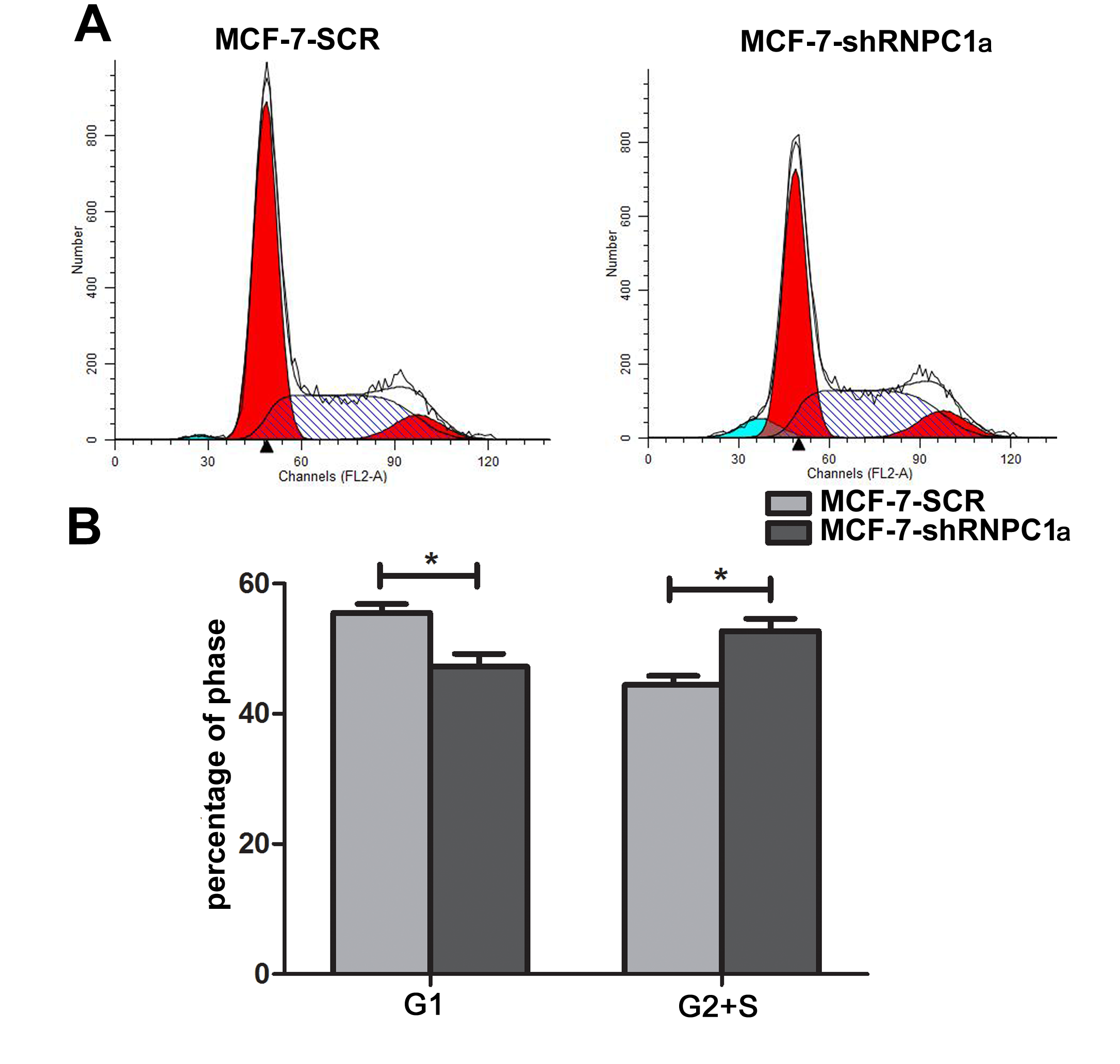

Supplement: Additional file 2: Figure S2 — Cell cycle was progress in RNPC1a knockdown MCF-7 cells. (A) The progression of MCF-7-SCR cells was more arrested in the G1 phase compared to MCF-7-shRNPC1a cells. (B) Histogram of cell cycle analyses. Data were means of three separate experiments mean ± SEM, *p < 0.05. [file 1471-2407-14-322-S2.tif]

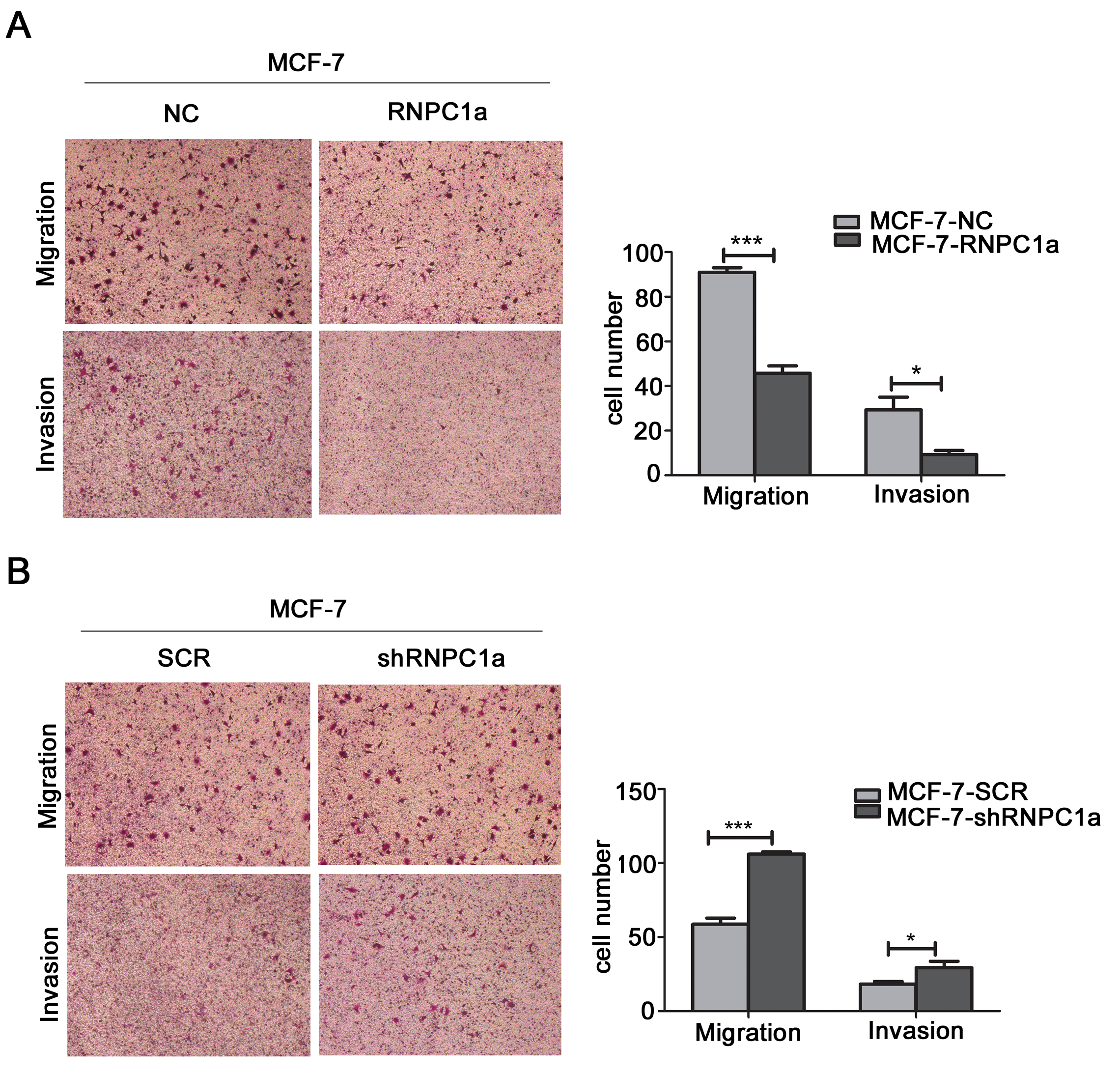

Supplement: Additional file 3: Figure S3 — RNPC1a decreased migration and invasion in MCF-7 cells. (A) The number of migrating and invading cells was higher in MCF-7-NC than the MCF-7-RNPC1a cells. (B) The number of migrating and invading cells was lower in MCF-7-SCR than the MCF-7-shRNPC1a cells. Data presented average number of cells/field for three fields. (C, D) Columns: average data of three independent experiments, mean ± SEM, *p < 0.05, ***p < 0.001. [file 1471-2407-14-322-S3.tif]
