# Supplementary material for: RNA-binding protein RNPC1: acting as a tumor suppressor in breast cancer
Source: BMC Cancer. 2014 May 7;14:322. doi: 10.1186/1471-2407-14-322 (PMC4101826; doi:10.1186/1471-2407-14-322)
Supplement: Additional file 1: Table S1 — RNPC1a shRNA sequences. Figure S1. Identification of stably transfected MCF-7 and MB-231 cells. (A, C) Western blot was used to verify the efficiency of knockdown. The cells transduced with the three shRNAs and one control shRNA are designated as ‘sh1’, ‘sh2’, ‘sh3’ and ‘SCR’. RNPC1a-knockdown MCF-7 and MB-231 cells had 85% lower expression when compared with SCR cells. The fold change of RNPC1a is shown below each lane. Arbitrarily set at 1.0 in control cells. The intensity of the bands was determined using densitometric analysis. (B, D) qRT-PCR was used to detect RNPC1a expression. The results are similar to those seen in the Western blot analyses. Data were means of two separate experiments mean ± SEM, * p < 0.05. [file 1471-2407-14-322-S1.zip › Additional file 1/Table S1.doc]

**Table 1** RNPC1 shRNA sequences.

| Group | 5'-3' sequence |
| --- | --- |
| Sh1 | CGCCATGCACGGCTCGCAGAAGGACACCA |
| Sh2 | ACACTACCGACGCCTCGCTCAGGAAGTAC |
| Sh3 | GGCACCACTTTCGTGCAGTACCAGGCGCC |
